# Kleptoparasitism and Coexistence: Resource Competition Between Indian Leopards and Striped Hyenas

**DOI:** 10.3390/ani15060784

**Published:** 2025-03-10

**Authors:** Reuven Yosef, Swapnil Kumbhojkar

**Affiliations:** 1 Eilat Campus, Ben Gurion University of the Negev, P.O. Box 272, Eilat 88106, Israel; 2 Jhalana Wildlife Research Foundation, Gharkul Society, Ganeshmala, Sinhagad Road, Pune 411030, India; swapniljhalana@gmail.com

**Keywords:** interspecific competition, carnivore coexistence, resource utilization strategies, mesopredator interactions

## Abstract

In ecosystems where multiple carnivores coexist, interspecific interactions are crucial in shaping behavioral adaptations and resource utilization strategies. This study examines the competitive dynamics between Indian leopards (*Panthera pardus fusca*) and striped hyenas (*Hyaena hyaena*) in the Jhalana Reserve Forest, an urban-enclosed wildlife habitat in Jaipur, India. Using direct observations, citizen science contributions, and camera-trap data, we document kleptoparasitism and competitive exclusion instances where hyenas successfully outnumber leopards to gain access to food. Our findings indicate that hyenas exhibit an acute ability to locate leopard kills, often arriving within minutes of a leopard beginning to feed. Additionally, spatial constraints imposed by the reserve’s fencing create an ecological imbalance, as leopards can access external food sources while hyenas remain confined. We also discuss the potential consequences of supplementary feeding practices, which may influence predator behavior and interspecific interactions. This study highlights the importance of understanding competitive dynamics in fragmented landscapes to inform conservation strategies that promote coexistence.

## Simple Summary

This study explores how leopards and striped hyenas interact in the Jhalana Reserve Forest, a wildlife area surrounded by the city of Jaipur, India. By directly observing these animals and using camera traps, we found that hyenas often try to steal food from leopards. Hyenas are able to locate leopard kills quickly and sometimes bring other hyenas to help outnumber the leopard and take the food. The study also looks at how the fenced boundaries of the reserve create an imbalance, with leopards able to hunt outside the reserve, while hyenas are trapped inside. Additionally, the practice of feeding roadkill to the animals raises concerns about how it might change their natural behaviors. Understanding how these predators compete and coexist is important for creating better conservation strategies in urbanized areas.

## 1. Introduction

In ecosystems where multiple carnivores coexist, interspecific interactions play a crucial role in shaping behavioral adaptations and resource utilization strategies [1,2,3]. Byambasuren et al. [4] demonstrated how Eurasian lynx (*Lynx lynx*) modify their spatial use in response to snow leopards (*Panthera uncia*). Among such interactions, those between leopards (*Panthera pardus fusca*) and striped hyenas (*Hyaena hyaena*) are of particular interest due to their overlapping distributions and competitive dynamics [5]. Leopards and striped hyenas have broad distributions across Africa and Asia, frequently coexisting in regions with limited prey availability [6]. Leopards are solitary, ambush predators that primarily hunt medium-sized ungulates, whereas striped hyenas are predominantly scavengers, though they are also capable of hunting small vertebrates and birds. Competition between these species arises primarily from their shared reliance on carrion and instances of kleptoparasitism, where hyenas attempt to appropriate kills made by other apex predators, including leopards [7,8,9]. This competitive dynamic has been observed in various ecological settings, highlighting strategic differences in their foraging and hunting behaviors [5,7]. Dutta et al. [9], in their evaluation of temporal and spatial segregation among tigers (*Panthera tigris*), leopards, and striped hyenas, concluded that hyenas depend on tigers and leopards for resources and that their coexistence is facilitated through strategic adaptations in resource acquisition.

Previous research and field observations suggest that while leopards have a competitive advantage in direct encounters due to their superior strength and agility, striped hyenas utilize persistence and numerical superiority to outcompete leopards for access to resources [10]. In Rajasthan, camera trap footage has documented hyenas approaching leopard kills, often compelling the solitary predator to abandon its meal [11]. Additionally, interactions at shared water sources and overlapping hunting territories further demonstrate the competitive pressures between these species. Hyenas are known to track leopards from a distance, waiting for an opportune moment to challenge them for food [12]. This behavioral adaptation underscores the importance of energy conservation in predator–prey dynamics.

Beyond direct confrontations, leopards and hyenas influence each other’s behavior through avoidance strategies. Leopards frequently hoist their kills into trees to minimize the risk of scavenging, while hyenas use vocal communication and scent marking to establish dominance over key scavenging sites [13]. Such behaviors illustrate the intricate balance of competition between these species, driven by the need to maximize resource acquisition while minimizing risk. Additionally, evidence from scat analysis and GPS tracking indicates that both species adjust their movement patterns based on the presence of the other, further emphasizing the significance of their ecological interactions [12].

This study aims to present these interactions using both documented field observations and publicly available media sources [14,15].

Understanding the behavioral ecology of these carnivores is critical for conservation planning, particularly in regions experiencing increased human–wildlife conflict and habitat fragmentation. By analyzing the competitive dynamics between leopards and striped hyenas, this study contributes to a broader understanding of how mesopredators and apex predators coexist within shared landscapes and the implications for ecosystem stability.

We questioned how Indian leopards and striped hyenas interact in terms of competition for resources within the Jhalana Reserve Forest, and what are the implications of these interactions for their coexistence in an urbanized environment? We hypothesized that striped hyenas will exhibit kleptoparasitic behavior towards Indian leopards, utilizing their numerical advantage to outcompete leopards for food resources in the Jhalana Reserve Forest.

## 2. Materials and Methods

**Figure 1:**
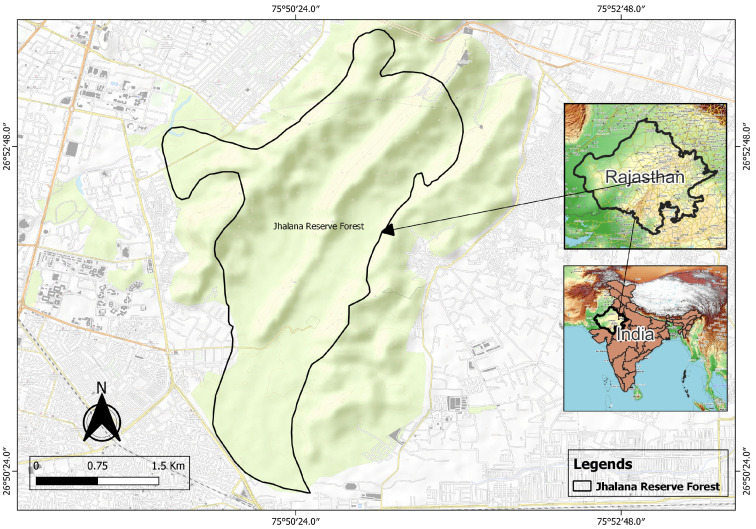
Map of Jhalana Reserve Forest, with its boundary outlined in black. The map indicates the relative location of Jaipur’s urban areas surrounding the reserve.

The Jhalana Reserve Forest, located in Jaipur, India, is a unique urban wildlife sanctuary spanning approximately 29 km^2^. Despite its relatively small size, the reserve supports a thriving population of leopards (*Panthera pardus fusca*) [16,17]) (Figure 1).

The reserve comprises a dry deciduous forest interspersed with rocky outcrops and sparse vegetation, providing an optimal habitat for leopards, which have adapted remarkably well to living in proximity to human settlements. Other species inhabiting the reserve include Indian foxes (*Vulpes bengalensis*), jungle cats (*Felis chaus*), and various ungulates, creating a dynamic ecosystem where predator–prey interactions are frequently observed [18].

The presence of striped hyenas in Jhalana adds an additional layer of ecological complexity, as they share overlapping territories with leopards. Although direct encounters between these species are relatively rare due to differing temporal activity patterns—leopards being primarily crepuscular and hyenas being largely nocturnal—competition for resources is ongoing. Spatial segregation has been observed (RY, SK, unpubl. data), with leopards typically denning among rocky cliffs, whereas striped hyenas predominantly inhabit dry streams.

**Figure 2:**
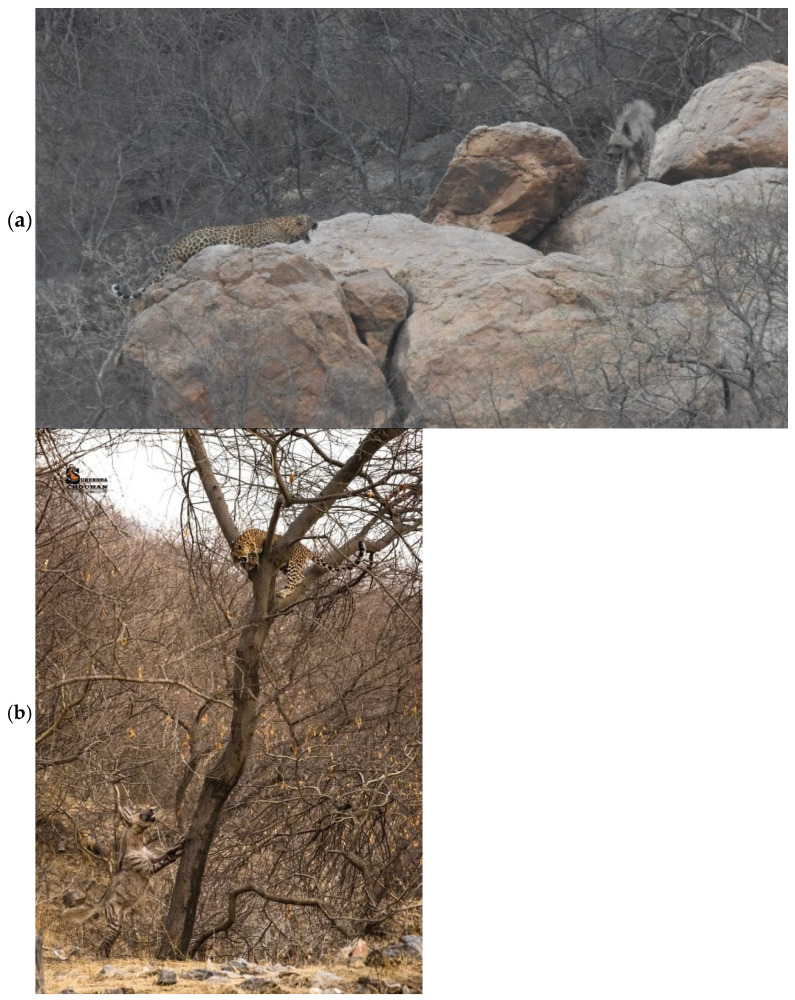
(**a**,**b**) Examples of leopard—striped hyena interactions in the Jhalana Reserve Forest. Photos: (**a**) Abhinav Mudgal, (**b**) Surendra Chouhan, Instagram (Surendra singh chouhan|Leopard And Hyeana Jhalana|Instagram (https://www.instagram.com/chouhan_photography_s/p/DFvSySGsyNq/) accessed 8 February 2025).

Nevertheless, numerous instances of direct interactions between leopards and striped hyenas have been documented in Jhalana by the authors (Figure 2a) and ecotourists (Figure 2b).

**Figure 3:**
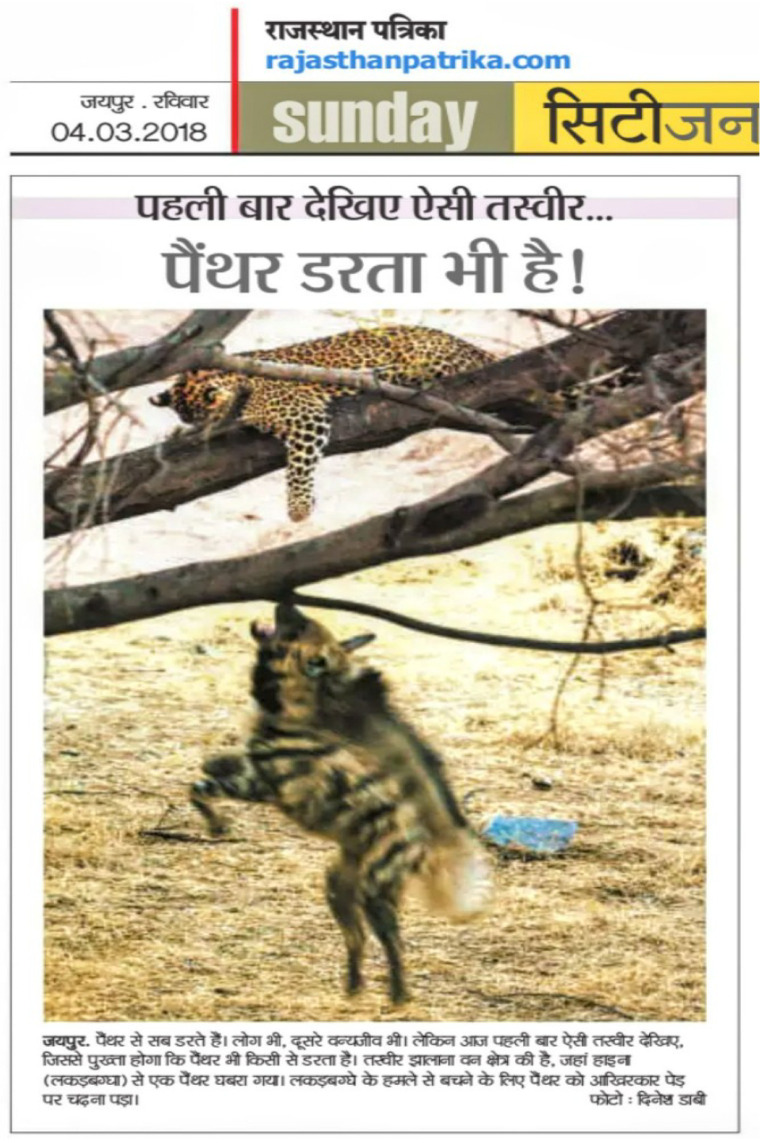
A published report on leopard–striped hyena interactions in local media (Rajasthan Patrika, 4 March 2018, Title: “Panthers are also afraid”).

As part of an ongoing research project on the leopards of Jhalana, we document predator interactions through personal observations, citizen science contributions, and 21 motion-sensor camera traps [16,18]. Additional encounters have also been recorded through public media platforms (Facebook, Instagram, YouTube, etc.), local television broadcasts, and newspapers (Figure 3).

We documented an incident in which a female leopard concealed the carcass of her cub—presumed to have been killed by a male—within a dense thicket of Thor cacti (*Euphorbia caudicifolia*) [19]. This behavior likely aimed to prevent scavengers, including striped hyenas, from feeding on the remains.

To address the limited availability of large prey species within the reserve, the Forest Service periodically collects roadkill from the surrounding areas and distributes it randomly within the forest to supplement food for carnivores. This practice is particularly significant for striped hyenas, which are confined within the reserve by a three-meter fence built atop a stone wall encircling the designated area. The fence was constructed to mitigate human–wildlife conflict, as Jaipur and neighboring villages border the reserve on nearly all sides [16]. However, while hyenas remain restricted within this enclosed space, leopards can scale the barrier with ease, granting them access to an additional prey base—stray dogs (*Canis lupus familiaris*)—in Jaipur’s urban environment [20].

Instances of kleptoparasitism have also been documented, in which hyenas scavenge leopard kills, forcing leopards to either defend their meal or abandon it to avoid injury. Several such incidents have been recorded using 21 motion-sensor camera traps (Cuddeback X-Change Color Model 1279, De Pere, WI, USA) deployed between November 2017 and June 2023 at carcasses brought by the rangers or regularly at the waterholes [18,20], and we present a sequence of events from one such occurrence here.

## 3. Results and Case Descriptions

Instances of 11 incidents of kleptoparasitism were documented, wherein hyenas scavenged leopard kills, compelling leopards to either defend their prey or abandon it to mitigate the risk of injury. Over this period, the camera traps captured 29,211 wildlife images, of which 382 (1.31%) depicted leopard–hyena interactions at carcasses. Additional interactions between these species were either directly observed and photographed by the researchers in the reserve or documented by ecotourists, who subsequently shared their observations on public media platforms.

Here, we present an incident documented on 10 January 2018, illustrating how striped hyenas gained access to a Nilgai (*Boselaphus tragocamelus*) carcass provided by forest authorities. The carcass was placed in an open area near a trail and was eviscerated to facilitate consumption by carnivores.

**Figure 4:**
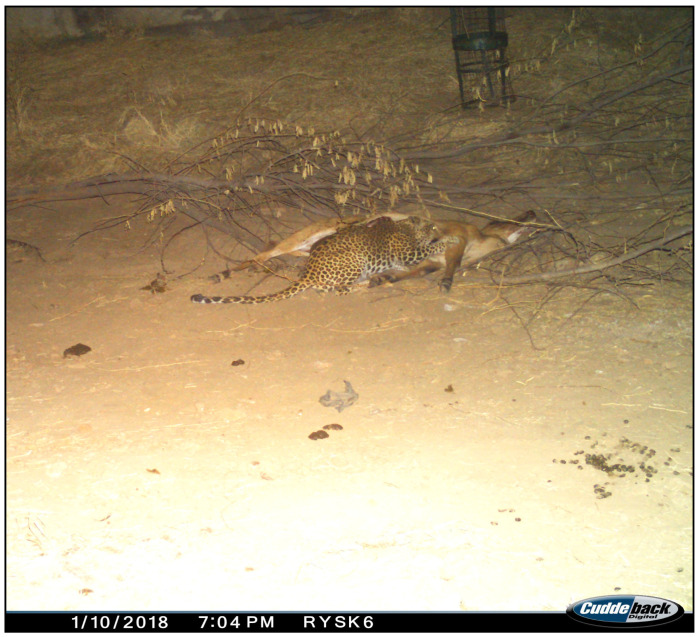
The leopard feeding at the Nilgai carcass.

**Figure 5:**
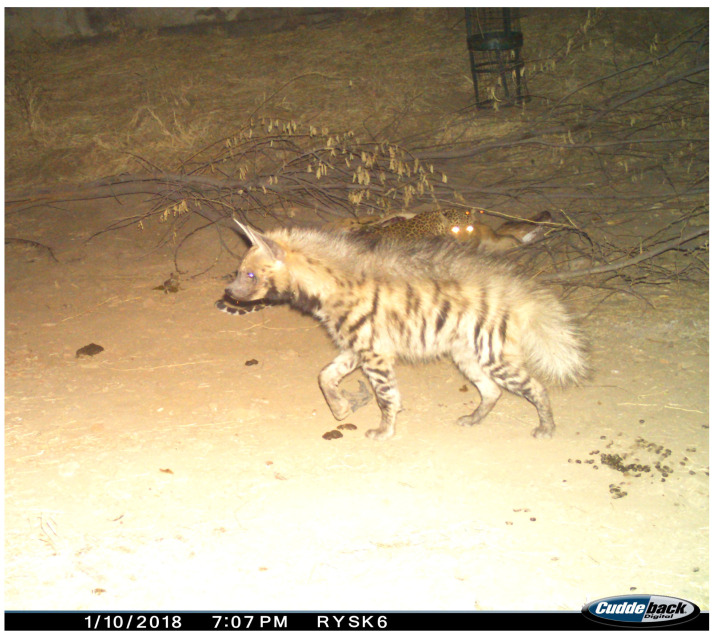
The initial approach of a striped hyena.

**Figure 6:**
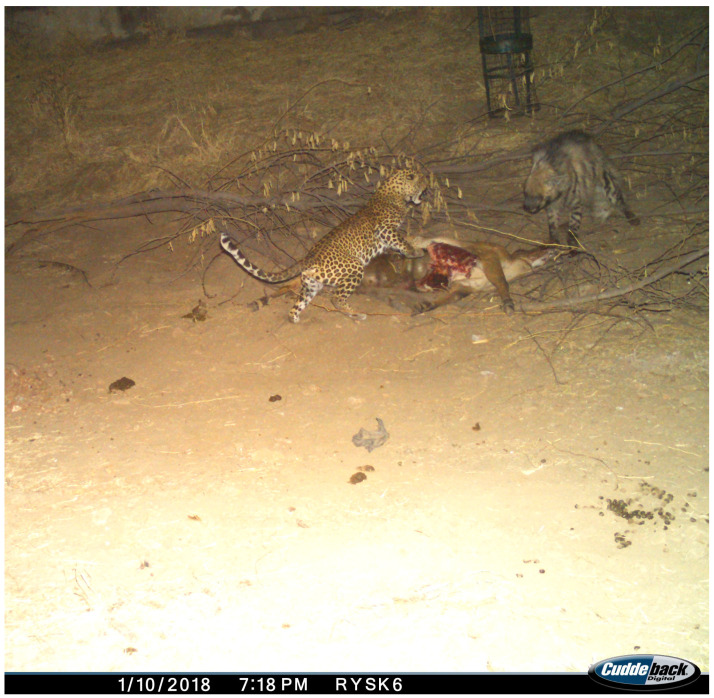
An aggressive defense of the Nilgai carcass by the feeding leopard.

A leopard discovered the carcass approximately an hour after its placement, at 19:00 h, and began feeding (Figure 4). Shortly afterward, at 19:07 h, a striped hyena arrived and attempted to access the carcass. However, the leopard aggressively defended its meal, preventing the hyena from feeding (Figure 5). The striped hyena persisted for nearly an hour, but each attempt was met with a hostile response from the leopard, forcing the hyena to retreat (Figure 6).

**Figure 7:**
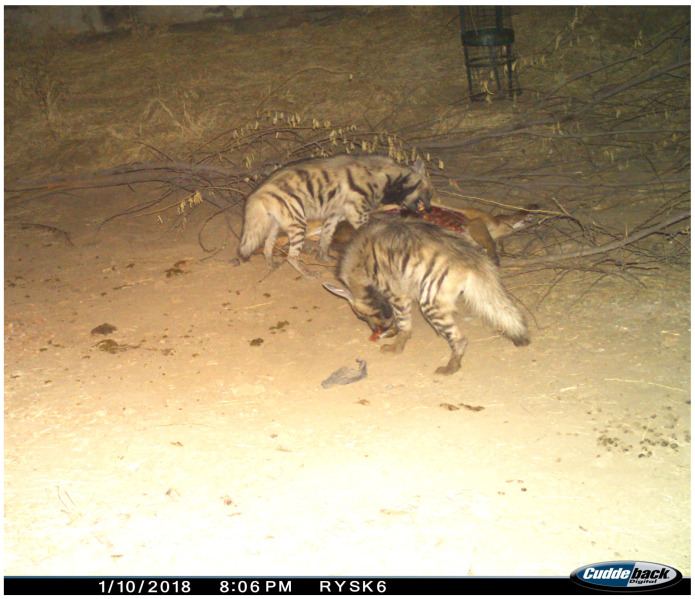
The subsequent take-over of the carcass by two striped hyenas.

At 19:55 h, the striped hyena temporarily withdrew but returned at 20:02 h, this time accompanied by another individual, presumed to be its mate. The presence of the second hyena altered the competitive dynamic; upon their approach, the leopard tore off a portion of meat from the shoulder region of the carcass and retreated, allowing the two striped hyenas to take possession of the remains (Figure 7).

**Figure 8:**
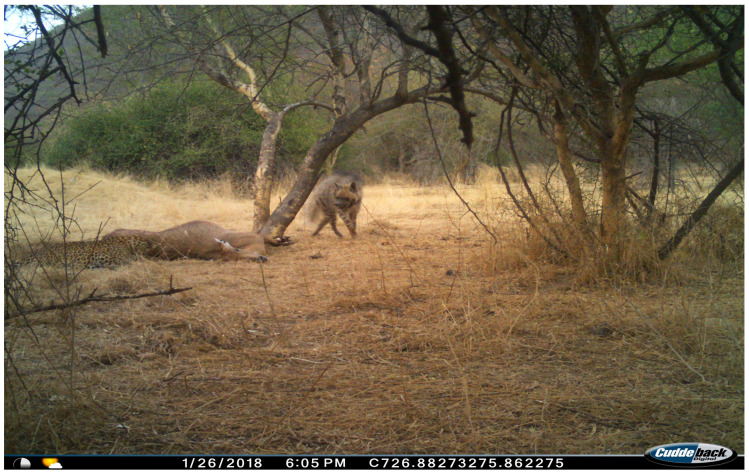
Another incident that occurred when striped hyenas accessed a carcass only when they outnumbered the feeding leopard.

A similar incident occurred on 26 January 2018, when a leopard feeding on a carcass (Figure 8) was discovered by a striped hyena. The primary difference between the two events was the latency of the hyena’s initial appearance. In the first incident, the hyena arrived merely four minutes after the leopard (19:03 vs. 19:07 h), whereas in the second case, it appeared nearly two hours later (16:16 vs. 18:05 h). The hyena ultimately secured access to the carcass by recruiting another individual, thereby outnumbering the leopard.

## 4. Discussion

This sequence of events highlights the competitive interactions between leopards and hyenas in Jhalana, where scavengers rely on persistence and social cooperation to secure food. Such observations offer valuable insights into interspecific interactions and resource partitioning in urban wildlife reserves.

The interactions between leopards and striped hyenas in the Jhalana Reserve Forest illustrate the competitive strategies these carnivores employ. They underscore the role of social behavior, persistence, and competitive exclusion in shaping predator dynamics. Understanding these dynamics is essential for conservation planning, particularly in areas facing increasing human–wildlife conflict [9,21]. Moreover, Jhalana’s status as a geophysically confined green island within an urban landscape presents unique ecological challenges that these predators must navigate.

A key factor influencing leopard–hyena interactions in Jhalana is the differential ability of these species to traverse the reserve’s fenced boundaries. While leopards can move beyond the enclosure, accessing alternative food sources, such as stray dogs in urban areas, hyenas remain confined within the reserve. This spatial restriction likely increases the hyenas’ reliance on scavenging within the reserve, intensifying competition over carcasses and leading to more frequent confrontations [9].

Our observations indicate that hyenas possess an acute ability to locate leopard kills and scavenging sites, often arriving at carcasses within minutes. Similar patterns have been widely documented in Africa [8,12]; however, our findings confirm that striped hyenas in Jhalana exhibit comparable behavior. This suggests a sophisticated cueing mechanism that allows hyenas to track leopard activity, particularly when a leopard is feeding.

In Africa, mammalian scavengers locate carrion twice as fast when following avian scavengers (e.g., vultures) that detect predator kills, such as those made by lions (*P. leo*) or cheetahs (*Acinonyx jubatus*). This exemplifies the significance of interspecific cueing in identifying carcasses [22,23,24]. However, beyond visual cues, other sensory mechanisms, such as auditory or olfactory signals, also play a role [23,25,26].

Notably, the Jhalana Reserve Forest lacks vultures, suggesting that visual cues may be less relevant in this environment. Further research is needed to determine whether striped hyenas primarily rely on olfactory cues, auditory signals, or learned behavioral patterns to locate carcasses.

Although kleptoparasitism and aggressive encounters between spotted hyenas (*Crocuta crocuta*) and leopards have been well documented in African ecosystems, there are also reports of unexpected instances of cooperation and prey sharing between these species, as seen in various media sources [27,28,29]. However, interactions between Indian leopards and striped hyenas remain underrepresented in scientific literature, despite the increasing documentation of such events by citizens and ecotourists [5,30]. Additionally, while hyenas are generally known to avoid areas frequented by leopards [3], our observations provide the first documented cases of striped hyenas successfully outnumbering a single leopard to gain access to a carcass.

Another important issue arising from this study is the impact of provisioning resources to predators. Supplementary feeding, whether intentional or unintentional, is widely considered detrimental, as it alters natural behaviors and ecological interactions [31,32]. Schmidt and Timm [33] reported that food provisioning is a major contributing factor to habituation, ultimately leading to increased human–wild canid conflicts. Behrendorff [34] further concluded that habituation often necessitates intervention, including the humane destruction of individuals in cases of severe conflict or aggression. In Jhalana, authorities frequently place roadkill within the reserve to supplement prey availability for predators confined within the urban-enclosed forest. While this practice may mitigate food shortages, its long-term ecological consequences remain unclear. Before continuing this intervention, authorities must conduct thorough assessments of its effects on predator behavior and interspecific interactions to prevent unintended disruptions to existing coexistence dynamics [16]. To mitigate interspecific competition and promote a balanced predator–scavenger dynamic in Jhalana, the Forest Department should adopt a more strategic approach to the dispersal of roadkill carcasses within the reserve. Proper spatial distribution of these food sources could reduce direct confrontations between leopards and hyenas while supporting their natural foraging behaviors [35]. Implementing feeding schedules aligned with the natural hunting and scavenging patterns of both species may further minimize conflict and encourage ecological stability.

To build on the findings of the current study, researchers could utilize GPS collars on both leopards and hyenas to track their movements and interactions in real time. This would provide valuable insights into spatial resource use and competitive dynamics [36]. Based on these analyses, reserve management strategies should be refined to include wildlife corridors that facilitate safe movement between key resource areas. Ensuring that reserve boundaries maintain access to critical resources—such as water sources and primary prey populations—would be essential to mitigating competition and fostering coexistence between these species.

Expanding the deployment of camera traps to monitor key locations where leopard–hyena interactions occur could provide further behavioral evidence of kleptoparasitism and competitive interactions, particularly in response to carcass availability. Establishing a long-term monitoring program is imperative to evaluate the effectiveness of these management strategies. Adaptive management approaches should be implemented, allowing for modifications based on observed outcomes—particularly if certain interventions inadvertently increase competition or alter natural behaviors.

Additionally, controlled scavenging trials could be conducted by strategically placing carcasses in different locations to assess scavenger response times and the effectiveness of various cueing mechanisms [37]. Sound playback experiments using recorded leopard vocalizations or feeding sounds, could further explore whether hyenas exhibit distinct behavioral responses to these auditory cues in controlled settings [38]. Ecological assessments should also be conducted to examine the broader impacts of supplementary feeding and habitat modifications on the predator–scavenger relationship.

Public participation in data collection could further enhance research efforts. The RFD should consider engaging ecotourists in documentation initiatives, encouraging them to share photographic evidence of leopard–hyena interactions. This citizen science approach could contribute valuable observational data, improving our understanding of the behavioral ecology of both species.

From a broader perspective, future research should explore the influence of urbanization and human–wildlife coexistence on leopard–hyena competitive dynamics in the Jhalana Reserve Forest. Investigating how supplementary feeding practices alter natural behaviors will be particularly valuable in guiding conservation strategies [39]. Ultimately, an improved understanding of interspecific interactions can inform conservation policies aimed at fostering coexistence between leopards and hyenas in increasingly urbanized landscapes. The conservation of leopards and striped hyenas in the Jhalana Reserve Forest is critical for maintaining ecological balance, as these apex predators regulate prey populations and contribute to overall biodiversity. Their presence serves as a key indicator of ecosystem health, reinforcing the need for targeted conservation efforts by the Forest Department. Beyond their ecological role, these species hold cultural significance and play a vital part in the local eco-tourism economy [40], making their protection beneficial not only for biodiversity but also for sustainable development. Effective management strategies should focus on mitigating human–wildlife conflict, ensuring habitat connectivity, and implementing research-driven conservation policies. Additionally, continued scientific investigations into their behaviors and interactions will provide valuable insights to enhance wildlife management practices. By prioritizing the well-being of leopards and hyenas, the resilience of the Jhalana Reserve Forest can be strengthened, ensuring the long-term sustainability of its ecosystem while promoting coexistence between wildlife and the surrounding human communities.

## 5. Conclusions

Our findings highlight the significance of interspecific competition in shaping predator behavior and resource utilization. The observed interactions between leopards and striped hyenas underscore the importance of conservation planning that accounts for the unique spatial and dietary needs of both species. Future research should focus on the long-term effects of competition on population trends and explore potential coexistence strategies that reduce direct confrontations. Additionally, further investigation into their adaptive behaviors and ecological roles will contribute to the long-term sustainability of predator populations in human-dominated landscapes.

## Data Availability

All data are included in the manuscript.

## References

[1] Reddy C.S., Yosef R., Calvi G., Fornasari L. (2019). Inter-specific competition influences apex predator–prey populations. Wildl. Res..

[2] Charaspet K., Pla-ard M., Paansri P., Keawdee B., Chanachai Y., Bhumpakphan N. (2021). Spatial and temporal overlaps of top predators: Dhole, tiger and leopard, and their potential preys in Huai Kha Khaeng Wildlife Sanctuary, Thailand. Biodiversitas.

[3] Ashish K., Ramesh T., Kalle R., Giordano A.J. (2024). Spatial heterogeneity facilitates coexistence between striped hyaenas and sympatric carnivores. J. Biogeogr..

[4] Byambasuren C., Johansson Ö., Alexander J.S., Lkhagvajav P., Samelius G., Sharma K. (2024). Who’s the boss? Understanding the spatial relationship between snow leopard and Eurasian lynx in southern Mongolia. Wildl. Biol..

[5] Panda D., Mohanty S., Allen M.L., Dheer A., Sharma A., Pandey P., Lee H., Singh R. (2023). Competitive interactions with dominant carnivores affect carrion acquisition of striped hyena in a semi-arid landscape of Rajasthan, India. Mammal Res..

[6] Davis R.S., Yarnell R.W., Gentle L.K., Uzal A., Mgoola W.O., Stone E.L. (2021). Prey availability and intraguild competition regulate the spatiotemporal dynamics of a modified large carnivore guild. Ecol. Evol..

[7] Ramesh T., Kalle R., Downs C.T. (2017). Staying safe from top predators: Patterns of co-occurrence and inter-predator interactions. Behav. Ecol. Sociobiol..

[8] Sloots M. (2021). Relationship Between Leopard and Spotted Hyena Using Camera Trap Data. https://www.wei.co.za/blog/relationship-between-leopard-and-spotted-hyena-on-a-wei-internship.

[9] Dutta S., Maheswaran G., Krishnamurthy R. (2024). Interspecific interactions among major carnivores in Panna Tiger Reserve: A multispecies occupancy approach. Biotropica.

[10] Vissia S. (2023). Competitive Co-Existence Within a Rich Large Carnivore Guild. Ph.D. Thesis.

[11] Gupta V.D., Areendran G., Raj K., Ghosh S., Dutta S., Sahana M. (2021). Assessing habitat suitability of leopards (*Panthera pardus*) in unprotected scrublands of Bera, Rajasthan, India. Forest Resources Resilience and Conflicts.

[12] Vissia S., Fattebert J., van Langevelde F. (2022). Leopard density and interspecific spatiotemporal interactions in a hyena-dominated landscape. Ecol. Evol..

[13] Balme G.A., Miller J.R., Pitman R.T., Hunter L.T. (2017). Caching reduces kleptoparasitism in a solitary, large felid. J. Anim. Ecol..

[14] Dylewski L., Tryjanowski P., Mikula P., Morelli F., Yosef R. (2017). Social media and scientific research are complementary—YouTube and shrikes as a case study. Sci. Nat..

[15] Hadad E., Yosef R. (2025). Unusual intra-specific aggression in Striped Hyena. Acta Ethologica.

[16] Kumbhojkar S., Yosef R., Benedetti Y., Morelli F. (2019). Human-leopard (*Panthera pardus fusca*) co-existence in Jhalana Forest Reserve, India. Sustainability.

[17] Wildlife Footage (168) Leopard and Hyena Interactions 2025, YouTube. https://www.youtube.com/playlist?list=PLrOSoZBmrGh2lOZqWbV-cFdGGp7XvhOlM.

[18] Kumbhojkar S., Mahabal A., Rakholia S., Yosef R. (2024). Avian and Mammalian Diversity and Abundance in Jhalana Reserve Forest, Jaipur, India. Animals.

[19] Yosef R., Dabi H., Kumbhojkar S. (2021). Thanatological behavior of a female Leopard (*Panthera pardus fusca*). Acta Ethologica.

[20] Kumbhojkar S., Yosef R., Kosicki J.Z., Kwiatkowska P.K., Tryjanowski P. (2020). Dependence of the Leopard *Panthera pardus fusca* in Jaipur, India, on domestic animals. Oryx.

[21] Doubleday K.F. (2020). Tigers and “good Indian wives”: Feminist political ecology exposing the gender-based violence of human–wildlife conflict in Rajasthan, India. Ann. Am. Assoc. Geogr..

[22] Mills M.G. (1990). Kalahari Hyaenas.

[23] Hunter J.S., Durant S.M., Caro T.M. (2007). Patterns of scavenger arrival at cheetah kills in Serengeti National Park Tanzania. Afr. J. Ecol..

[24] Kane A., Kendall C.J. (2017). Understanding how mammalian scavengers use information from avian scavengers: Cue from above. J. Anim. Ecol..

[25] Ogada D.L., Torchin M.E., Kinnaird M.F., Ezenwa V.O. (2012). Effects of vulture declines on facultative scavengers and potential implications for mammalian disease transmission. Conserv. Biol..

[26] Naves-Alegre L., Morales-Reyes Z., Sánchez-Zapata J.A., Sebastián-González E., Ovaskainen O. (2022). Scavenging in the realm of senses: Smell and vision drive recruitment at carcasses in Neotropical ecosystems. Proc. Royal Soc. B.

[27] Jazynka K. (2018). Leopard and Hyena Sharing a Kill in Sabi Sand Game Reserve. https://www.youtube.com/watch?v=gbgwC9fGL5w.

[28] National Geographic Wild Leopard & Hyena: Strange Alliance. https://www.youtube.com/watch?v=cFWuaimfcEc.

[29] Earth Touch News (2022). Rare Footage of Leopard and Hyena Sharing a Meal. https://www.youtube.com/results?search_query=Rare+Footage+of+Leopard+and+Hyena+Sharing+a+Meal.

[30] Mandal D., Chatterjee D., Qureshi Q., Sankar K. (2018). Behavioural observations on interaction of leopard and striped hyena, western India. Cat News.

[31] Newsome T., van Eeden L.M. (2017). The effects of food waste on wildlife and humans. Sustainability.

[32] Newsome T.M., Dellinger J.A., Pavey C.R., Ripple W.J., Shores C.R., Wirsing A.J., Dickman C.R. (2014). The ecological effects of providing resource subsidies to predators. Glob. Ecol. Biogeogr..

[33] Schmidt R.H., Timm R.M. Bad dogs: Why do coyotes and other canids become unruly?. Proceedings of the 12th Wildlife Damage Management Conference Proceedings.

[34] Behrendorff L. (2021). Best-practice dingo management: Six lessons from K’gari (Fraser Island). Aust. Zool..

[35] Moleón M., Sánchez-Zapata J.A., Selva N., Donázar J.A., Owen-Smith N. (2014). Inter-specific interactions linking predation and scavenging in terrestrial vertebrate assemblages. Biol. Rev..

[36] Tarugara A., Clegg B.W., Gandiwa E., Muposhi V.K. (2021). The effect of competing carnivores on the feeding behaviour of leopards (*Panthera pardus*) in an African savanna. Ecol. Evol..

[37] Beasley J.C., Olson Z.H., DeVault T.L., Benbow M.E., Tomberlin J.K., Tarone A.M. (2015). Ecological role of vertebrate scavengers. Carrion Ecology, Evolution and Their Applications.

[38] Hettena A.M., Munoz N., Blumstein D.T. (2014). Prey responses to predator’s sounds: A review and empirical study. Ethology.

[39] Thompson C. (2024). The Human-Wildlife Interface: A Case Study of Bobcat & Coyote Conflict in California. Master’s Thesis.

[40] Lucas C.A.C. (2022). Evaluating Human-Carnivore Coexistence Using a Multi-Stakeholder Socio-Ecological Approach. Ph.D. Thesis.

